# Temperature-Sensitive Reproduction and the Physiological and Evolutionary Potential for Mother’s Curse

**DOI:** 10.1093/icb/icz091

**Published:** 2019-06-07

**Authors:** Kristi L Montooth, Abhilesh S Dhawanjewar, Colin D Meiklejohn

**Affiliations:** School of Biological Sciences, University of Nebraska–Lincoln, 1104 T Street, Lincoln, NE 68502, USA

## Abstract

Strict maternal transmission of mitochondrial DNA (mtDNA) is hypothesized to permit the accumulation of mitochondrial variants that are deleterious to males but not females, a phenomenon called mother’s curse. However, direct evidence that mtDNA mutations exhibit such sexually antagonistic fitness effects is sparse. Male-specific mutational effects can occur when the physiological requirements of the mitochondria differ between the sexes. Such male-specific effects could potentially occur if sex-specific cell types or tissues have energy requirements that are differentially impacted by mutations affecting energy metabolism. Here we summarize findings from a model mitochondrial–nuclear incompatibility in the fruit fly *Drosophila* that demonstrates sex-biased effects, but with deleterious effects that are generally larger in females. We present new results showing that the mitochondrial–nuclear incompatibility does negatively affect male fertility, but only when males are developed at high temperatures. The temperature-dependent male sterility can be partially rescued by diet, suggesting an energetic basis. Finally, we discuss fruitful paths forward in understanding the physiological scope for sex-specific effects of mitochondrial mutations in the context of the recent discovery that many aspects of metabolism are sexually dimorphic and downstream of sex-determination pathways in *Drosophila*. A key parameter of these models that remains to be quantified is the fraction of mitochondrial mutations with truly male-limited fitness effects across extrinsic and intrinsic environments. Given the energy demands of reproduction in females, only a small fraction of the mitochondrial mutational spectrum may have the potential to contribute to mother’s curse in natural populations.

## Introduction

### The evolutionary potential for a female-specific selective sieve on mitochondrial mutations

Strict maternal inheritance of the mitochondrial genome (mtDNA) has the potential to function as a sex-specific selective sieve, in which the fate of mitochondrial mutations is governed solely by their selective effects in females (Lewis 1941; Frank and Hurst 1996; Gemmell et al. 2004). Mitochondrial mutations that are neutral in females but deleterious in males can fix in a population by drift, while sexually antagonistic mutations that benefit females and are deleterious in males will be fixed by positive selection. Frank and Hurst (1996) offered this population genetic explanation, later termed mother’s curse (Gemmell et al. 2004), for the observation that some human diseases linked to mitochondrial mutations tend to disproportionately affect males.

If, as population genetics dictates, natural selection is powerless to halt the decline of male-specific mtDNA functions, what prevents species from extinction due to mtDNA-linked male sterility? The hypothesis that has received the most attention proposes that compensatory evolution at nuclear loci restores male function (Frank 1989; Frank and Hurst 1996; Ågren et al. 2019). Mitochondrial replication, transcription, translation, and oxidative phosphorylation (OXPHOS) require hundreds of gene products encoded in the nuclear genome that, in theory, could reverse mother’s curse. Predicting the significance and dynamics of a female-specific selective sieve on mitochondrial mutations therefore requires understanding the population genetics of interactions between alleles at mtDNA and nuclear loci (hereafter mito–nuclear interactions). Theory predicts that mito–nuclear polymorphisms will rarely be maintained in populations (Clark 1984; Gregorius and Ross 1984), with the exception of X-linked alleles, as the coupled transmission of the X chromosome and the mtDNA in females can sustain mito–nuclear fitness variation, particularly when this variation is sexually antagonistic (Rand et al. 2001). Supporting this theory, work in the fruit fly *Drosophila* has shown that X chromosomes from a single population generate sexually antagonistic fitness variation when combined with different mtDNAs (Rand et al. 2001; Montooth et al. 2010).

If male-detriment mtDNAs fix in populations, then recovery of male fitness could require subsequent evolution at nuclear-encoded loci. Simulations predict that the male-specific mtDNA mutational load arising from this dynamic could be substantial and exceed mutational load across the nuclear genome (Connallon et al. 2018), and compensatory nuclear alleles that alleviate the effects of mother’s curse mutations are predicted to be enriched on the Y chromosome (Ågren et al. 2019). However, population and transmission genetic parameters such as the degree of heteroplasmy, inbreeding, and even low levels of paternal mtDNA transmission can reduce the scope for a female-specific mtDNA selective sieve. For example, moderate inbreeding, where sisters mate with brothers with whom they share a mtDNA, is predicted to reverse mother’s curse by exposing mutations with deleterious effects in males to natural selection (Unckless and Herren 2009; Wade and Brandvain 2009). Thus, while male-detrimental mutations in mtDNA can potentially accumulate in populations, this potential may vary across populations and species as a function of population genetic parameters such as the level of inbreeding. Nonetheless, across all populations the prevalence of male-detriment mtDNA polymorphisms will ultimately depend on the scope for mtDNA mutations to have male-specific or sexually antagonistic fitness effects.

### Empirical support for a female-specific selective sieve

Direct empirical support for a female-specific selective sieve that permits the accumulation of male-deleterious mitochondrial mutations comes from the discovery of mitochondrial polymorphisms with male-specific effects segregating within populations. Male-sterile mtDNAs have been recovered from both wild and mutagenized *Drosophila* (Clancy 2008; Xu et al. 2008; Clancy et al. 2011; Patel et al. 2016). Quantitative effects of mtDNAs on male fertility and sperm competition have been also been measured in fruit flies (Yee et al 2013; Camus and Dowling 2018). Male-sterile mtDNAs can segregate cryptically in populations if their sterility effects are masked by nuclear alleles that restore male function (Clancy 2008; Clancy et al. 2011), a dynamic that has been well documented to underlie cytoplasmic male sterility in plants (Lewis 1941; Frank 1989; Budar et al. 2003; Delph et al. 2007; Case et al. 2016). However, mtDNA variants that affect only males may be the exception, and their discovery and characterization in animals have thus far been limited to a handful of organisms, including *Drosophila*. In the marine copepod *Tigriopus californicus*, mito–nuclear interactions compromise both female and male reproductive fitness in inter-population hybrids (Willett 2008). In humans, the evidence supporting stronger effects of mitochondrial disease mutations in males versus females is sparse (Beekman et al. 2014). While mitochondrial mutations were originally implicated in human male infertility with no detected effects in females (Moore and Reijo-Pera 2000; Ruiz-Pesini et al. 2000), these findings have been called into question, and it remains unclear the extent to which male infertility is associated with mitochondrial haplotype (Mossman et al. 2012). Even for human mitochondrial diseases where male-biased effects have been documented (e.g., Leber’s Hereditary Optic Myopathy), it is not clear whether this is caused by interactions with recessive X-linked alleles that are exposed in hemizygous males or because of male-specific effects of the mitochondrial mutations (Chinnery and Schon 2003; Beekman et al. 2014). Thus, while mtDNA variants with the sex-specific selective effects required for mother’s curse do exist, whether they are exceptional or the norm remains an open question (Dowling and Koch 2019, this issue).

Several patterns of genome evolution have been proposed as indirect evidence of mother’s curse. In *Drosophila*, many nuclear-encoded mitochondrial genes are part of gene families, with evolutionarily young paralogs that are expressed solely in testes (Gallach et al. 2010). These gene duplication events are hypothesized to reverse mother’s curse by providing a nuclear target for mutations with effects that are restricted to males and can be acted on by selection (Gallach and Betrán 2011). Although this model is intriguing, to date no evidence beyond the existence and expression pattern of these duplicates supports or refutes their role in reversing mother’s curse. Indeed, this pattern is not evident in humans (Eslamieh et al. 2017), and there is no molecular signature of recurrent positive selection on male-specific OXPHOS paralogs in *Drosophila* (Havird 2019, this issue)—a signature that might be expected if molecular changes at these gene duplicates restored male fitness. Additionally, the *Drosophila* genome harbors many evolutionarily young paralogs with testis-specific expression that do not have mitochondrial function (Belote and Zhong 2009; White-Cooper 2010). A model of nuclear restoration of sexually antagonistic effects of mtDNA mutations has also been proposed to favor the movement of nuclear genes with mitochondrial function off of X chromosomes, which spends two-thirds of their time in females and are co-transmitted with the mtDNA (Drown et al. 2012). However, this pattern of chromosomal distribution of nuclear genes with mitochondrial function is taxonomically limited (Drown et al. 2012; Dean et al. 2014; Hough et al. 2014; Ågren et al. 2019) and could be driven by other phenomenon involving sex chromosomes such as dosage compensation. Thus, it remains unclear the extent to which mother’s curse mutations accumulate in natural populations and generally impact genome evolution.

### Sex-specific effects of a model mito–nuclear incompatibility in *Drosophila*

Sex-specific and sexually antagonistic fitness effects of mtDNA mutations may result from 1) sex-specific effects on physiology and development that impact fitness or 2) sex-specific fitness consequences of mtDNA mutations with similar physiological effects in males and females. The fruit fly *Drosophila melanogaster* has emerged as a powerful model for manipulating natural mtDNA and nucDNA variation to test for the presence of sexually antagonistic effects on life-history traits related to fitness (Rand 2001; Montooth et al. 2010; Camus et al. 2012, 2015; Camus and Dowling 2018). Here we synthesize results from studies dissecting a model mito–nuclear incompatibility in *Drosophila* across different levels of biological organization to understand its potential to generate sex-specific fitness effects.

**Fig. 1:**
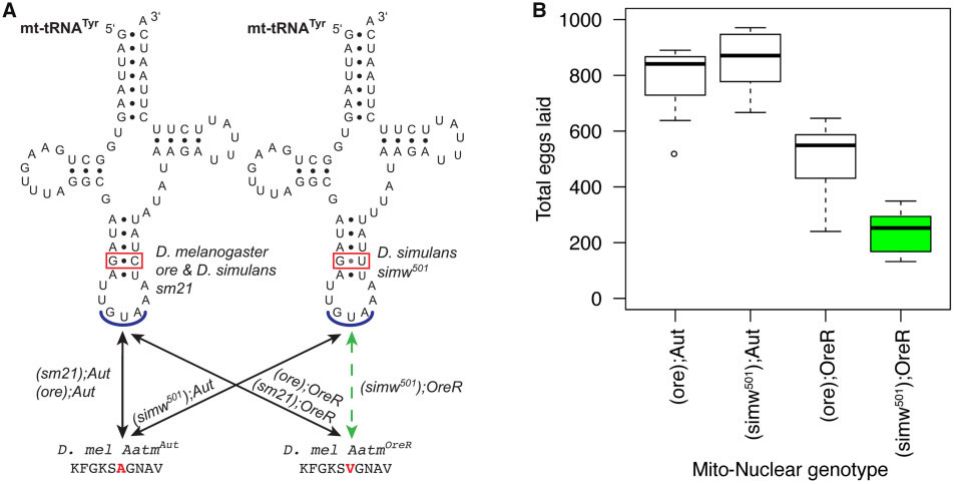
A model of mito–nuclear incompatibility in fruit flies arises from interactions between a mtDNA polymorphism in the anticodon stem of the mt-tRNA^Tyr^ and a nuclear amino acid polymorphism in the mitochondrially targeted aminoacyl tRNA^Tyr^ synthetase that charges the mt-tRNA^Tyr^ for mitochondrial protein synthesis. **A**) The mitochondrial and nuclear SNP genotypes of the six strains used in this study. The (*simw^501^*); *OreR* genotype combines incompatible mitochondrial and nuclear SNPs, while the other (*mtDNA*); *Nuclear* genotypes serve as genetic controls. Figure modified from Hoekstra et al. (2013) with permission from the Genetics Society of America. **B**) Females with the mito–nuclear incompatible genotype, highlighted in green, lay significantly fewer eggs at 25°C. The *y*-axis is total eggs laid by a female over 10 days. The figure is from Hoekstra et al. (2018).

Mito–nuclear coevolution to maintain mitochondrial function is predicted to result in the accumulation of mito–nuclear incompatibilities between divergent populations and closely related species. We have previously identified, mapped, and functionally characterized a specific mito–nuclear incompatibility between a mitochondrial polymorphism in the mt-tRNA^Tyr^ from *Drosophila simulans* and a nuclear amino acid polymorphism in the mitochondrial-targeted tyrosine aminoacyl tRNA synthetase protein from *D. melanogaster* (Fig. 1). Each polymorphism has little to no effect on its own, but the combination of these polymorphisms in individuals with the (*simw^501^*); *OreR* genotype produces a suite of deleterious effects due to compromised mitochondrial protein synthesis. Adult male and female flies with the (*simw^501^*); *OreR* genotype have decreased activity of OXPHOS complexes that require mitochondrial protein translation (Complexes I, III, IV, and V of the electron transport chain), relative to control genotypes (Meiklejohn et al. 2013). Embryonic survival is compromised in this genotype, but surviving embryos have normal larval and pupal mortality, although larval development is severely delayed and this delay is exacerbated in environments that normally accelerate growth and increase energy demand (Hoekstra et al. 2013, 2018; Buchanan et al. 2018). The ability to grow and accumulate the energy stores required for metamorphosis in spite of compromised OXPHOS activity is enabled by compensatory upregulation of the TCA cycle, glycolytic ATP production, and respiration rates in larvae with this mito–nuclear incompatible genotype (Hoekstra et al. 2013; Matoo et al. 2019).

The compensatory physiological changes that facilitate development in larvae with the mito–nuclear incompatible genotype are associated with fitness costs later in life. Relative to genetic controls, larvae with this mito–nuclear incompatibility accumulate greater levels of hydrogen peroxide, a reactive oxygen species, and have lower mitochondrial membrane potentials (Matoo et al. 2019). We hypothesize that the lower membrane potential may result from mitochondrial uncoupling as a physiological defense mechanism to prevent additional free radical production (Matoo et al. 2019). While adults with this mito–nuclear incompatibility have normal metabolic rates, they suffer a number of other defects associated with fitness. They have smaller and more brittle sensory bristles (Meiklejohn et al. 2013), decreased male and female survival when infected with a natural bacterial pathogen (Buchanan et al. 2018), and compromised female fecundity (Meiklejohn et al. 2013; Zhang et al. 2017; Hoekstra et al. 2018).

Females with the mito–nuclear incompatibility suffer reduced fecundity at 25°C that is exacerbated by stress. Females with the (*simw^501^*); *OreR* genotype that survive bacterial infection have significantly decreased fecundity relative to their sham-infected sisters, revealing a potential trade-off between immunity and fecundity that is not observed in control genotypes (Buchanan et al. 2018). Females with the (*simw^501^*); *OreR* genotype that develop at 28°C are sterile, while control genotypes maintain fertility at this temperature. The temperature-dependent sterility in females with the (*simw^501^*); *OreR* genotype results from the combined effects of compromised ovarian development, loss of germline stem cells, and possible underprovisioning of embryos (Zhang et al. 2017). Males have normal fecundity at 25°C (Hoekstra et al. 2018), but our inability to culture this mito–nuclear genotype at 28°C when using either female or males of this genotype as parents suggested that there may also be temperature-dependent male sterility caused by this mito–nuclear incompatibility (Hoekstra et al. 2013). While 28°C is far from critical thermal maxima in adult *D. melanogaster* (Hoffmann 2010; Sgrò et al. 2010), development at 28°C is significantly accelerated and nearer to thermal limits (∼32°C) for robust egg-to-adult viability (Petavy et al. 2001). We have hypothesized that this increased rate of development may place significant demand on organismal energy supply systems (Hoekstra et al. 2018).

Here we report new experiments that revealed a temperature-dependent effect of the mito–nuclear incompatibility on male fertility, similar to that previously observed in females. Temperature-dependent sterility in males with the mito–nuclear incompatibility is due to defects in spermatogenesis rather than in sperm function at high temperatures and can be partially rescued by diet, as might be expected if the sterility had an energetic basis.

## Materials and methods

We used six genotypes that combined mtDNA from *D. melanogaster* (mtDNA: *ore*) and *D. simulans* (mtDNA: *sm21, simw^501^*) with two inbred, wild-type nuclear genomes from *D. melanogaster* (nucDNA: *OreR* and *Aut*), and included the (*simw^501^*); *OreR* mito–nuclear incompatible genotype. These genotypes enabled us to test for phenotypic effects of an interaction between genetic variation in the mtDNA and nuclear genomes (Fig. 1). The *sm21* mtDNA differs from the *ore* mtDNA at over 600 nucleotide sites, but differs from the *simw^501^* mtDNA at only six sites in the coding region of the mtDNA that include the tRNA mutation that causes the mito–nuclear incompatibility (Meiklejohn et al. 2013; Fig. 1). When the phenotypes of individuals with the *sm21* mtDNA and *ore* mtDNA are similar, but differ from individuals with the *simw^501^* mtDNA and the *OreR* nuclear background, this suggests that the characterized mito–nuclear interaction underlies the phenotypic effect.

All genotypes were maintained at a permissive room temperature (∼22°C). Twenty-five males and 25 females from each genotype were allowed to mate and oviposit for 6 h at 25°C and then were removed from the vials. All genotypes were robustly fertile when developed at 25°C (Buchanan et al. 2018; Hoekstra et al. 2018). Eggs hatched and larvae developed at 25°C until the early third-instar, when they were transferred to 28°C. Upon eclosion, five males from each genotype were individually housed with three virgin females from an outbred population. The outbred population was generated by combining isofemale genetic strains—two from Ithaca, NY, two from the Netherlands, and three from Zimbabwe. Females of this outbred population were robustly fertile at 22–28°C and used for all experiments. Experimental males were transferred to new vials with three new virgin females every 2 days, and the old vials were moved to 25°C to minimize the effect of temperature on the development of any offspring. This was repeated five times over a 10-day period, giving each experimental male ample opportunity to demonstrate fertility. Fertility for each male was measured as the average number of offspring sired per vial to account for males who died before the end of the 10 days. Because offspring inherited their mtDNA and half of their nuclear alleles from their outbred mothers, differences in the number of offspring can be attributed to fertility of the paternal genotype, and not effects of the mito–nuclear incompatibility on offspring survival.

To test for an effect of diet, we used a standard maltose diet and three approximately isocaloric diets that differed in their protein:carbohydrate ratios (High P:C = 452 kJ/100 g, 7.1% protein, 17.9% carbohydrate; Equal P:C = 456 kJ/100 g, 4.3% protein, 21.2% carbohydrate; Low P:C = 469 kJ/100 g, 2.5% protein, 24.6% carbohydrate; values from Matzkin et al., 2011). The relative abundances of protein and carbohydrates were achieved by altering the ratio of yeast:sucrose in the diet (Table 1). All genotypes were raised on the experimental diets for at least two generations before the fertility trials. We repeated these experiments across three blocks for a sample size of 15 males per genotype per diet. The average reproductive output for each genotype on each diet was calculated by first averaging the number of offspring sired by each male across the number of time points the male was alive, and then averaging across the 15 replicate males. The data were analyzed using linear models that tested for the fixed effects of mtDNA, nuclear genome, diet, and the interactions between these factors.

**Table 1:** Isocaloric diets varied in protein:carbohydrate (P: C) via differences in yeast:sucrose content (based on diets from Matzkin et al., 2011)

Ingredient	Standard diet	High P:C diet	Equal P:C diet	Low P:C diet
Agar (g)	1.86	1	1	1
Torula yeast (g)	16.6	32	20	8
Cornmeal (g)	20	9	9	9
Sucrose (g)	–	8	20	32
Molasses (mL)	9.3	–	–	–
Tegosept (g)	0.6	0.45	0.45	0.45
95% ethanol (mL)	3.3	4.5	4.5	4.5
Propionic acid (mL)	1.3	–	–	–
Distilled water (mL)	200	200	200	200

## Results

### Temperature-dependent male sterility effects of a mito–nuclear incompatibility

**Fig. 2:**
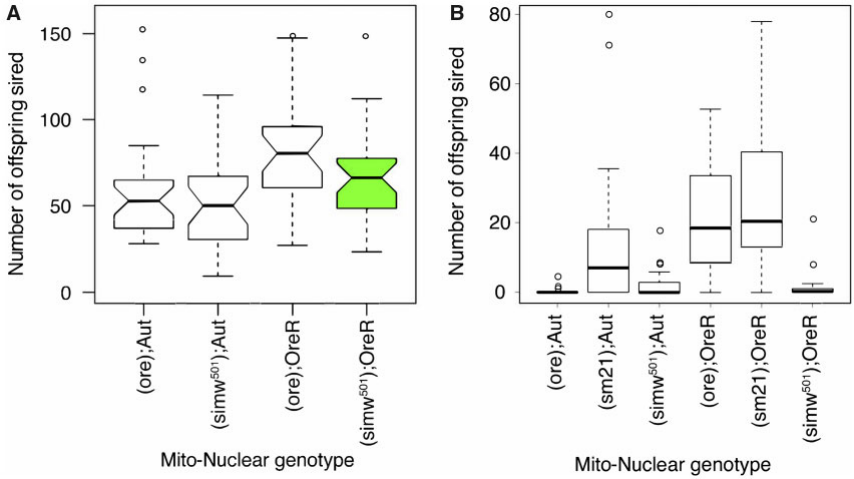
Males with the mito–nuclear incompatible genotype, highlighted in green, have wild-type fertility at 25°C, but are sterile at 28°C. **A**) When males were developed at 25°C, there was no evidence that mito–nuclear genotype affected male offspring production. The *y*-axis is the number of offspring sired by individual males divided by the number of females (up to three) that produced offspring after 48 h of mating, averaged across 28–29 replicate males of each genotype. Figure from Hoekstra et al. (2018). **B**) When males were developed at 28°C, there was a significant mito–nuclear interaction effect on fertility, with (*simw^501^*); *OreR* males producing almost no offspring. The *y*-axis is the number of offspring sired by individual males that were given two new females every 2 days for 10 days, averaged across 15 replicate males of each genotype.

**Fig. 3:**
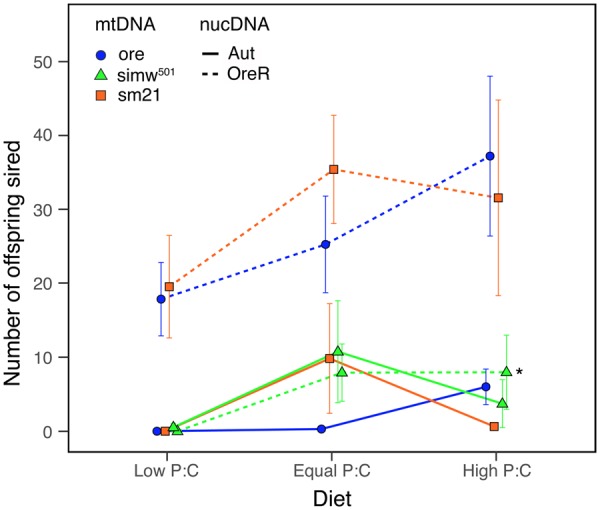
The protein:carbohydrate (P:C) ratio affects temperature-dependent sterility in males reared at 28°C on three approximately isocaloric diets. Increasing P:C in the diet was able to rescue some fertility in males of genotypes that were sterilized by development at 28°C, including males with the (*simw^501^*); *OreR* mito–nuclear incompatible genotype, highlighted with an asterisk. The *y*-axis is the number of offspring sired by individual males that were given two new females every 2 days for 10 days, averaged across 15 replicate males of each genotype. Error bars are standard errors.

At 25°C, males with the mito–nuclear incompatibility did not have compromised fertility and there was no significant effect of the mito–nuclear interaction on fertility (Fig. 2A;Hoekstra et al. 2018). However, when males were developed at 28°C there was a significant effect of the mito–nuclear interaction on male fertility (Table 2; mtDNA × nuclear, *P *<* *0.0001). Males with the (*simw^501^*); *OreR* incompatibility had greatly reduced fertility, relative to their nuclear genetic controls (Fig. 2B). This temperature-dependent loss of fertility was not due to impaired sperm function at 28°C; males developed at 25°C and mated at 28°C had robust fertility (Table 3). Supporting the hypothesis that this temperature-sensitive effect on male fertility has an energetic basis, diet modified both fertility generally (diet, *P *=* *0.019) and modified the effects of the mtDNA (mtDNA × diet, *P *=* *0.025) (Table 4). Increasing protein relative to carbohydrates in the diet tended to have a positive effect on fertility, and also produced the largest rescue of temperature-dependent sterility in males with the mito–nuclear incompatibility (Fig. 3).

**Table 2:** Analysis of variance of genetic effects on the number of offspring sired by males developed at 28°C on the standard diet

Factor	χ^2^	Num DF	*P*-value
mtDNA	43.652	2	<0.0001
nucDNA	30.513	1	<0.0001
mtDNA:nucDNA	20.482	2	<0.0001

**Table 3:** The proportion of males that sired more than 100 offspring when reared at 25°C and transferred to 28°C for mating on the standard diet

Genotype^a^	Days 1–2	Days 3–4	Days 5–6	Days 7–8	Days 9–10
(*sm21*); *Aut*	1	1	0.71	0.14	0.14
(*sm21*); *OreR*	1	1	0.625	0.25	0
(*ore*); *Aut*	1	0.56	0.29	0	0
(*ore*); *OreR*	0.9	0.67	0.75	0.125	0
(*simw^501^*); *Aut*	0.9	0.67	0.56	0	0
(*simw^501^*); *OreR*	1	0.9	0.56	0	0

a Males of experimental genotypes and females from an outbred wild-type population were reared at 25°C and transferred to 28°C for mating. Individual males were housed with three virgin females for 2 days, and then transferred to new vials with three new virgin females, for a total of 10 days. After males were removed from the vials, mated females and eggs were returned to 25°C and vials were scored for fertility.

**Table 4:** Analysis of variance of genetic and diet effects on the number of offspring sired by males developed at 28°C on three approximately isocaloric diets

Factor	χ^2^	Num DF	*P*-value
mtDNA	24.817	2	<0.0001
nucDNA	114.242	1	<0.0001
Diet	7.955	2	0.0187
mtDNA:nucDNA	52.633	2	<0.0001
mtDNA:Diet	11.168	4	0.0247
nucDNA:Diet	0.159	2	0.9233
mtDNA:nucDNA:Diet	3.377	4	0.4967

A striking pattern in this model system is the strong sexually antagonistic effect of the nuclear genotype on female and male reproductive fitness. Females with the *Aut* nuclear genotype have much higher fecundity relative to females with the *Ore* nuclear genotype (Fig. 1B; Meiklejohn et al. 2013; Hoekstra et al. 2018). In contrast, males with the *Aut* nuclear genotype have lower fertility than males with the *Ore* nuclear genotype (Fig. 2A;Hoekstra et al. 2018). Males with the *Aut* nuclear genotype were also particularly thermally sensitive, showing almost complete sterility when developed at 28°C (Fig. 2B). Thus, male flies with the *Aut* nuclear background were sterile at 28°C due to effects of the nuclear background, while male flies with the (*simw^501^*); *OreR* incompatibility are sterile at 28°C due to the mito–nuclear interaction in this genotype. In contrast, females with the (*ore*); *Ore* genotype did not show temperature-dependent sterility (Zhang et al. 2017). Thus, temperature appears to magnify the sexually antagonistic effect of the nuclear genome, but through temperature-dependent sterility in males rather than females.

## Discussion

We found that an incompatible mito–nuclear genotype generates sterility in both sexes as a result of exposure to elevated temperatures during larval development. While there may be differences in the underlying causes of female and male sterility in this system, suggesting some scope for sex-specific physiological outcomes in gametogenesis, the broader pattern is that the compromised energetics caused by this mito–nuclear genotype negatively impacts both female and male reproduction when energy demand exceeds supply. In females, demand appears to exceed supply even at normal developmental temperatures. In males, reproductive capacity is compromised when demand exceeds supply during development and spermatogenesis at higher temperatures.

### Moving forward: the physiological potential for a female-specific selective sieve

The shared role of mitochondria in females and males to supply energy via OXPHOS would seem to provide limited scope for sex-specific effects of mtDNA mutations on physiological performance and fitness. However, recent advances in developmental biology reveal that there may be substantial sexual dimorphism in metabolism and development of organs that have historically been viewed as sexually monomorphic (Millington and Rideout 2018). For example, gut physiology and intestinal stem cell proliferation differs between the sexes, is influenced by juvenile hormone, and can affect lipid metabolism and adult reproductive output (Reiff et al. 2015; Hudry et al. 2016). Given the role of the gut in nutrient absorption and energy homeostasis, sexual dimorphism in gut physiology and development may cause genetic variation in energy metabolism to have different physiological consequences in males and females. Some of the sexual dimorphism in fruit fly physiology and development is directly regulated by sex-determination genes (Hudry et al. 2016; Sawala and Gould 2017; Millington and Rideout 2018; Rideout et al. 2015). For example, activity of the sex-determination gene *transformer* in the larval fat body regulates differences in body size between males and females via insulin signaling (Rideout et al. 2015), which influences rates of larval growth that establish sexual dimorphism in adult body size (Okamoto et al. 2013; Testa et al. 2013; Sawala and Gould 2017). Particularly relevant to mitochondrial function, males and females differ in their oxidative stress biology (Pomatto et al. 2017). Sexual dimorphism established early in development and persisting in adult organ homeostasis and cellular maintenance has clear potential to guide investigation of the physiological potential for mitochondrial mutations to have sex-specific effects.

The genetically and developmentally independent processes of oogenesis and spermatogenesis may lend themselves to sex-specific effects of mtDNA mutations, particularly if one of these processes tends to be more sensitive to mutations affecting energy metabolism. Frank and Hurst (1996) suggested that male reproduction may be particularly susceptible to the effects of mtDNA mutations; for example, in mammals, mitochondrial function is thought to provide energy for swimming sperm. However, the extent to which glycolysis and OXPHOS share the labor of ATP production is an active area of investigation in mammals (Mukai and Okuno 2004; du Plessis et al. 2015) and appears to vary among the insects that have been studied (Werner and Simmons 2008). A division of labor proposed by du Plessis et al. (2015) in which OXPHOS-generated ATP is used for mammalian sperm development, maturation, and some forms of motility, while glycolysis-generated ATP is used for hyperactivated motility, capacitation and the acrosome reaction, is consistent with our findings in *Drosophila*; male flies with the mito–nuclear incompatibility that developed at 25°C produce sperm that function at 28°C, while males that develop at 28°C, a temperature that enhances the OXPHOS energetic defect (Hoekstra et al. 2013), develop sperm that do not successfully fertilize females even when those fertilized females are placed at 25°C. Female reproduction is also energetically costly; a meta-analysis across a wide range of animal taxa estimated that, on average, females have a gamete biomass production rate that is approximately two to four orders of magnitude higher than males (Hayward and Gillooly 2011), suggesting that the energy demands of gametogenesis are usually greater in females than in males. In both male and female *Drosophila*, gametogenesis is sensitive to energy and nutrient availability, and restricting dietary protein slows germline cell proliferation which is reversible upon restoring dietary protein in both sexes (Drummond-Barbosa and Spradling 2001; McLeod et al. 2010; Yang and Yamashita 2015). Thus, while gametogenesis may be the most likely aspect of animal biology to show sex-specific effects of mtDNA mutations, it remains unclear whether males will more often be negatively impacted than females. The significance of the mother’s curse hypothesis ultimately rests on the proportion of mtDNA mutations that affect male reproductive fitness with no deleterious effects in females.

Even if mitochondrial variation has similar effects on male and female physiology or gamete production, this variation could nonetheless generate sexually antagonistic fitness effects if males and females differ significantly in their life history or ecology. For example, sexual dimorphism in dispersal patterns, in the intensity of intrasexual competition for resources, territories, or mates, and in the energetic investment in life-history traits, can all potentially impact the extent to which female or male fitness is determined by genetic variation in energy metabolism. An energetic framework that considers energy supply–demand balance in the context of development, physiology, life history, and ecology may be a powerful approach to predict where on the tree of life we expect significant accumulation of male-detrimental mitochondrial mutations that make it through the female-specific selective sieve. mtDNA and mito–nuclear genetic effects are often environmentally sensitive (Willett and Burton 2003; Hoekstra et al. 2013; Zhu et al. 2014; Mossman et al. 2016; Patel et al. 2016), and extrinsic and intrinsic conditions that elevate energy demand are expected to reveal effects of mutations that compromise energy supply (Hoekstra et al. 2018). An important question is whether mtDNA mutations with male-specific effects remain male-specific across a range of developmental stages and ecological conditions. Thus, integrative physiology and eco-physiology have an important role to play in our growing understanding of mito–nuclear evolutionary and ecological dynamics.
